# Ramipril-Induced Annular Bullous Pemphigoid: A Clinical Mimic of Linear IgA Dermatosis

**DOI:** 10.5152/eurasianjmed.2026.251053

**Published:** 2026-01-13

**Authors:** Zeynep Karaca Ural

**Affiliations:** Department of Dermatology, Atatürk University Faculty of Medicine, Erzurum, Türkiye

To the Editor,

Bullous pemphigoid (BP) is an autoimmune, subepidermal blistering disorder caused by antibodies targeting structural proteins of the epidermal basement membrane.^1^ Common risk factors include advanced age, neurologic disorders (e.g., dementia, Parkinson’s disease), and specific medications such as loop diuretics, spironolactone, and neuroleptics.^2^ Dysregulated T cell responses and IgG/IgE autoantibodies against BP180 and BP230 play a central role by triggering neutrophil chemotaxis and basement membrane degradation.^3^ Clinically, BP presents with a broad spectrum of manifestations, ranging from localized to generalized pruritic bullae. Notably, 20% of patients may lack bullous lesions entirely, displaying only excoriations, urticarial plaques, or prurigo-like lesions.^2^^,^^3^

Annular BP is a rare variant with lesions that may clinically resemble linear IgA bullous dermatosis. To our knowledge, drug-induced annular BP has not been previously reported. Herein, we present a case of annular BP associated with ramipril.

A 76-year-old male presented with pruritic bullous lesions that initially appeared on his back 9 months prior and later localized to the legs. His only comorbidity was hypertension, treated with ramipril 5 mg daily, which he had started four weeks before symptom onset. Systemic review was unremarkable. Physical examination revealed tense bullae and annular erythematous plaques with vesicular rings on both legs. Blood tests showed elevated total IgE (407 ng/mL; normal: 1.31-165) and eosinophils (1.02 × 10^3^/μL; normal: 0.01-0.47 × 10^3^). Linear IgA bullous dermatosis was considered due to the annular morphology, prompting a punch biopsy and direct immunofluorescence (DIF).

Histopathology revealed subepidermal separation, dermal edema, dense eosinophilic infiltrates, and eosinophilic exocytosis. The DIF demonstrated linear C3 deposition (++). These findings, in combination with clinical and laboratory data, supported a diagnosis of annular BP. Given the timing of lesion onset and the absence of alternative causes, ramipril was suspected as the trigger. The drug was discontinued, and amlodipine was prescribed as a replacement. Treatment with topical 0.05% clobetasol propionate and oral antihistamines was initiated.

At 3-month follow-up, all lesions had resolved, leaving only postinflammatory hyperpigmentation. No new eruptions occurred with continued topical therapy. Written informed consent was obtained from the patient before the case was presented.

The BP has numerous subtypes—including localized, pretibial, nodular, dyshidrosiform, vegetans, and annular forms—many of which can mimic other dermatoses such as nummular eczema, sarcoidosis, or mycosis fungoides.^4^ In our patient, the annular configuration closely resembled linear IgA dermatosis.

While only 15% of BP cases have an identifiable trigger, potential inducers include infections, physical agents, and drugs—especially in genetically predisposed individuals. These agents may modify the immune response or alter the antigenic properties of the basement membrane.^5^ Ramipril has been reported among medications associated with BP, and our patient had not used any other drugs. The resolution of symptoms following its withdrawal supports its role as a likely trigger.

This case underscores the importance of detailed medical history in patients with atypical bullous presentations. Clinicians should consider medications such as ramipril as possible culprits and discontinue them when appropriate.

## Data Availability

The data that support the findings of this study are available on request from the corresponding author.
